# Mitochondrial DNA, a Powerful Tool to Decipher Ancient Human Civilization from Domestication to Music, and to Uncover Historical Murder Cases

**DOI:** 10.3390/cells8050433

**Published:** 2019-05-09

**Authors:** Maxime Merheb, Rachel Matar, Rawad Hodeify, Shoib Sarwar Siddiqui, Cijo George Vazhappilly, John Marton, Syed Azharuddin, Hussain AL Zouabi

**Affiliations:** Department of Biotechnology, American University of Ras Al Khaimah, Ras Al Khaimah 10021, UAE; rachel.matar@aurak.ac.ae (R.M.); rawad.hodeify@aurak.ac.ae (R.H.); shoib.siddiqui@aurak.ac.ae (S.S.S.); cijo.vazhappilly@aurak.ac.ae (C.G.V.); jmarton@aurak.ac.ae (J.M.); azharuddin.syed@aurak.ac.ae (S.A.); hussain.zouabi@aurak.ac.ae (H.A.Z.)

**Keywords:** mitochondrial DNA, ancient DNA, ancient trade routes, domestication, ancient leather, ancient glue, ancient human diet, mummies, burnt human remains, Louis XVII, Tsar Nicholas II, Erard, Beethoven

## Abstract

Mitochondria are unique organelles carrying their own genetic material, independent from that in the nucleus. This review will discuss the nature of mitochondrial DNA (mtDNA) and its levels in the cell, which are the key elements to consider when trying to achieve molecular identification in ancient and degraded samples. mtDNA sequence analysis has been appropriately validated and is a consistent molecular target for the examination of biological evidence encountered in forensic cases—and profiling, in certain conditions—especially for burnt bodies and degraded samples of all types. Exceptional cases and samples will be discussed in this review, such as mtDNA from leather in Beethoven’s grand piano, mtDNA in mummies, and solving famous historical criminal cases. In addition, this review will be discussing the use of ancient mtDNA to understand past human diet, to trace historical civilizations and ancient trade routes, and to uncover geographical domestication origins and lineage relationships. In each topic, we will present the power of mtDNA and how, in many cases, no nuclear DNA was left, leaving mitochondrial DNA analysis as a powerful alternative. Exploring this powerful tool further will be extremely useful to modern science and researchers, due to its capabilities in providing us with previously unattainable knowledge.

## 1. Introduction

Mitochondria are dynamic and essential organelles that are found in almost all eukaryotic cells, and their main function is Adenosine triphosphate (ATP) production by oxidative phosphorylation. In addition, mitochondria are involved in lipid and amino acid metabolism and play important roles in various cellular processes, such as cell proliferation, apoptosis, and cell differentiation [1]. Mitochondria are organelles that have their own genomic DNA [2], where each mitochondrion is estimated to contain one to ten mitochondrial DNA copies [3]. Many studies have confirmed that several thousand copies of mitochondrial DNA can be found in just one animal cell [4,5]. Human mitochondrial DNA is a circular molecule of around 16,600 YBP, which encodes 13 structural polypeptide components required for oxidative phosphorylation. Translation of those polypeptide genes occurs in the mitochondrial matrix by a unique and very specific mechanism. In addition, mtDNA encodes 22 transfer RNAs and 2 ribosomal RNAs [6,7]. Exclusive maternal inheritance of mtDNA is observed in many sexually reproducing species. This pattern of a non-Mendelian manner of inheritance has been explained by several possible mechanisms, such as reduction of the amount of mtDNA during spermatogenesis or its elimination from mature spermatozoa. Other reasons for the loss of mtDNA in the zygote include post-fertilization selective degradation of the paternal mtDNA, or even the whole paternal mitochondrion [1,8]. Another possible mechanism for that paternal mtDNA withdrawal is simply by preventing sperm mitochondria from entering the oocyte [1]. Structural and biochemical analyses suggest that mtDNA replication happens by strand displacement. In this model, mtDNA replication of the leading (heavy) strand starts, initiating at a specific site called OH in the non-coding control region called the D-loop. When leading strand replication has reached around two-thirds of the genome, the origin of replication (OL) on the lagging (light) strand is exposed, allowing l-strand synthesis to occur in the opposite direction. 

Assessment of DNA survival in the ancient remains started in 1984 with Higuchi and his colleagues, in the Department of Biochemistry at University of California [9]. In this first ancient DNA analysis attempt, Higuchi’s team examined dried muscle of the quagga, a zebra-like species (*Equus quagga*) that became extinct in 1883 [9]. Although the DNA was a low molecular weight, with just 1% of the amount that would be expected from fresh muscle, Higuchi and his colleagues were able to obtain many clones from the quagga DNA, from which two pieces containing mitochondrial DNA were sequenced [9]. Despite the technical difficulties of ancient DNA studies, the 1984 quagga publication encouraged many researchers to conduct ancient DNA studies in other different ancient remains, and most of those studies were focused on mitochondrial DNA. Due to the low DNA recovery rate from ancient remains, mitochondrial DNA is always the best choice to target, as sequences from multi-copy loci have more chance to be detected in ancient remains than single-copy sequences, due to the presence of larger numbers per cell [10]. With reference to the aforementioned characteristics of mitochondrial DNA, we can assume that this genome would be the best target in ancient and degraded remains for the following reasons: (i) mtDNA is especially useful because it is present at a high copy number in cells [11]. (ii) It is more likely to survive for prolonged periods compared to nuclear DNA. (iii) Because of its unique maternal inheritance, mtDNA is also very useful in forensic identification cases [12,13] and for determining maternal family relationships when a gap of several generations exists between an ancestor and a descendant [14]. In this review, we will shed light upon the many ways that mtDNA is a useful and successful tool to investigate and solve historical cases, identify evidence in burnt human remains, solve rape and murder cases, and determine the source of remains in unsolved mysteries, especially in mummies and ancient music sound. In addition, this review discusses the use of ancient mtDNA to understand past human diets, to trace historical civilizations and ancient trade routes, and to uncover geographical domestication origins and lineage relationships.

## 2. mtDNA in Famous Historical Cases and Burnt Human Remains

mtDNA has been proven to be a powerful tool with which to reveal the mysteries associated with many historical murder cases. Many old cases have been either re-investigated or re-opened with the help of DNA profiling, which enabled strengthening of the evidence against the suspect or identification of new leads and new suspects. Current standard methods based on short tandem repeats (STRs) as well as lineage markers (Y chromosome, mitochondrial DNA) have further helped to expand the knowledge for crime investigators [15,16]. A few historical cases wherein mtDNA was successfully used in such circumstances are discussed below.

### 2.1. The Dottie Cox Mystery

Human remains with fractured skull and bones were recovered from a construction site in Danforth Avenue in Toronto, Ontario, in 1995 [17]. The skeletal analysis revealed a female who might have died under unusual circumstances several decades ago. On the basis of facial reconstruction, a Torontonian called Ronald Cox claimed that the picture greatly resembled photographs of his mother, Dottie Cox, who disappeared in 1943. Initial DNA analysis from her bone and teeth matched with the DNA from the sons of Dottie Cox (Ronald and Melville Cox). The analysis was conducted in Dr. David Sweet’s laboratory at the University of British Columbia, where he used STR analysis. In his report, Dr. Sweet indicated that the sons’ DNA matched that recovered from the Danforth Avenue unknown remains. In addition, he stated that the inclusion was based on mtDNA analysis. Since Dr. Sweet’s lab did not conduct mtDNA analysis, this automatically raised questions. Later, detailed examinations proved that, although blood and buccal samples of Ronald and Melville Cox were identical, as they shared four polymorphic differences, their samples failed to match with Danforth Doe’s bone skeletal DNA [17]. These experiments were carried out at Molecular World Inc., a private mtDNA testing facility in Thunder Bay, by Arlene Lahti and Curtis Hildebrandt. The experimental results clearly confirmed that there was no matching of the mtDNA from Danforth Avenue remains with the mtDNA of the sons. This case study further warranted reevaluation of the reliability of data obtained from mtDNA testing centers, especially in old cases.

### 2.2. The Truth about Louis XVII’s Death

The truth about Louis XVII’s death in the Temple Prison of Paris during the French Revolution on June 8, 1795 has been in question since the official declaration that said it was caused by tuberculosis. There were many rumors and theories, and one of the strongest legends said that he had been taken away or escaped out of the kingdom, and that his body had actually been substituted by that of another 10 year old boy [18]. A few decades later, several men claimed to be the son of Louis XVII, one of whom was Karl Wilhelm Naundorff, who was buried in The Netherlands. An interesting study by Jahaes et al. [19] concluded that Naundorff’s claim was not true, as mtDNA of Naundorff’s bone did not match with the mtDNA of Queen Anna of Romania and her brother André de Bourbon-Parme, who both are maternal relatives of Louis XVII. Two years later, another study was conducted by the same team of Jahaes et al. [18] to answer the two hundred year old question: Did Louis XVII die along with his family, or was he able to escape outside the kingdom? To find the answer, the heart of Louis XVII, preserved in a crypt at the Basilique Saint-Denis in Paris, was used by the second study of Jahaes and his colleagues. The tradition has always been to preserve the hearts of the dead members of the royal family, so this sample was attainable. Due to this study, the mystery was finally solved and the rumors were rejected, as the mtDNA of the previously analyzed maternal samples matched with the mtDNA of Louis XVII’s heart.

### 2.3. The Mystery of the Last Royal Family in Russia

Official records regarding the fate of the Romanov Imperial Family (The last Russian Royal family) remain incomplete and mysterious to this day. In July 1918, Tsar Nicholas II, his wife, Alexandra, and their five children (Olga, Tatiana, Maria, Anastasia, and Alexei) were held captive in the cellar of the Ipatiev House at Ekaterinburg, Russia, where they were eventually executed by a Bolshevik firing squad, putting an end to the House of Romanov dynasty that had lasted for over three centuries [20]. In the mid-1970s, Geli Ryabov and Alexander Avdonin, two Russians who were intrigued by the history of the Romanovs, began investigating the archived materials and photographs. They found an indication that led to the discovery of a shallow grave containing the Romanovs’ skeletons [21], but it was concluded that two bodies were missing, specifically the Tsarevich Alexei and one of the daughters (thought to be Anastasia) [22]. In 1992, Gill et al. [20] were approached by the Chief Forensic Medical Examiner of the Russian Federation with a request to initiate an official investigation to verify the authenticity of the remaining parts utilizing DNA based techniques. The research team followed a dual strategy, using STR analysis to conclude whether a family group was present, and mtDNA analysis to determine whether or not the bodies were maternally related descendants of the Romanov family. HRH Prince Philip, the Duke of Edinburgh, is a grand-nephew of unbroken maternal descent from Tsarina Alexandra. He provided Gill et al. [20] a sample of blood for comparison. mtDNA analysis allowed Gill et al. to confirm that the children were siblings, and the mother was identified among the nine skeletons that were found. In addition, all of the mtDNA sequences of the children and the identified mother matched with the mtDNA sequence from the Duke of Edinburgh. Another comparison was made using the mtDNA sequence of the Tsar, along with two relatives of unbroken maternal descent from Tsar’s maternal grandmother. The two relatives matched the sequence of the Tsar, except for one nucleotide at position 16169. Upon additional examination, it was determined that heteroplasmy at this position contained nucleotides C and T present at a ratio of approximately 3.4:1. Experiments were conducted utilizing cloning, which indicated that this mixture was due to heteroplasmy from the Tsar, although the ‘mismatch’ created a controversy that has lingered regarding the authenticity of these remains. The lack of convincing DNA evidence has kept the official final report on the fate of the Romanovs delayed by Russian authorities. Another study, conducted two years later on the request of the Russian government, was done by the Engelhardt Institute of Molecular Biology, Russian Academy of Sciences, Moscow. The institute analyzed the skeletal remains of the Tsar’s brother, Georgij Romanov, to gain further insight into the occurrence and segregation of heteroplasmic mtDNA variants in the Tsar’s maternal lineage. The mtDNA sequence results of Georgij Romanov were phenomenal, as they matched with those of Tsar and were heteroplasmic at the same position. This confirms heteroplasmy in the Tsar’s lineage, and also presents powerful evidence that identifies Tsar Nicholas II. The rapid intergenerational shift from heteroplasmy to homoplasmy and the different heteroplasmic ratios in the brothers is consistent with a ‘bottleneck’ mechanism of mtDNA segregation [14]. Doubts pertaining to the authenticity of the remains persisted [23] despite all the overwhelming forensic evidence. The two children missing from the mass grave—Alexei and one of his sisters—were pointed out by skeptics as being hard evidence that the bodies found did not indeed belong to the Romanov family. Shortly after the revelation of the first mass grave, a few endeavors in the following years were made to locate the second grave, which was strongly believed to be nearby [24]. In the summer of 2007, a group of archeologists found a few bone fragments roughly 70 meters from the first grave. Following an official archeological excavation, a set of 44 bone fragments and teeth were cautiously recovered from the site [24]. In 2009, two impartial laboratories highly specialized in ancient DNA (aDNA) studies, the Armed Forces DNA Identification Laboratory (AFDIL, Rockville, Maryland, USA) and the Institute for Legal Medicine (GMI, Innsbruck, Austria), reported a forensic DNA study led by Coble et al. on the remains discovered in 2007, using mtDNA, autosomal STR and Y-STR analysis [24]. In this study, Coble et al. presented additional DNA testing of material from the 1991 grave attributed to Tsar Nicholas II, his wife Alexandra, Olga, Tatiana, and a third daughter who ought to be either Anastasia or Maria. Coble et al. results indicated that the two individuals recovered from the 2007 grave are the two missing children of the Romanov family: Tsarevich Alexei and one of his sisters.

## 3. Ancient Music Sound Restoration through Mitochondrial DNA

Sebastian Erard, the famous piano maker, has produced various grand pianos, owned by many historical composers such as Joseph Haydn and Ludwig van Beethoven [25,26,27]. Although over two hundred and fifty (250) stunning pianos were made by Erard, only a few are in existence today, and it is stated that none of the pianos preserves its original sound. As an example, the Linz museum in Austria has Beethoven’s grand piano, which has been subjected to multiple alterations resulting in modification of the sound [27]. Among over 7500 instruments and art objects in the Museum of Music in Paris, the unique grand piano created by Erard in the 1800s has been preserved in its original state. A fascinating study was published not long ago aiming to restore Beethoven’s music by utilizing the same materials in the original grand piano. The study revolved around the use of mitochondrial DNA as a unique investigating tool. Sebastian Erard always covered the hammer head with leather for better sound quality. Merheb et al. [27] wanted to identify the species origin of this leather during their study. Three separate extractions were performed, collecting three samples containing a mixture of glue and leather. Unfortunately, the mixture could not be separated. However, in the same hammer, the authors found an isolated drop of glue, with which they expected to irrefutably identify the species used in the making of the glue. After considerable suggestions from experts in Citéde la Musique in Paris regarding possible species used in leather manufacture in the XVIII and XIX centuries, Merheb et al. began to amplify the mtDNA extracts with primers specific for the following species: sheep, cattle, goat, pig, and chamois. The first complication after sequencing and sequence identification was the presence of sheep mtDNA and cattle mtDNA in both leather and glue. While the extracted drop of glue had no contact with the leather, from these results it can be concluded that the glue had been made from sheep and cattle tissues. Normally, natural piece leather is produced from the hide of only one animal; thus, a single species should be identified. Therefore, the connection used to conclude the origin of the isolated glue sample could not be applied in the cases of the leather sample, where both cattle mtDNA and sheep mtDNA were detected. Quantitative polymerase chain reaction (Q-PCR) experiments were performed on each extract, then the ratio *R* = amount of the cattle mtDNA divided by the amount of sheep mtDNA was calculated. This means that, within the leather samples, if the leather fraction did not deliver mtDNA, the result would show that the latter had solely been issued from the glue fraction. Thus, we should acquire an equivalent ratio *R* in both leather and glue extracts. However, results showed that ratio *R* was way higher in leather than in glue. Mathematically, the ratio *R* increases if cattle mtDNA increases or sheep mtDNA decreases. This last possibility was not considered by the authors, as eventual PCR inhibition cannot happen selectively in the same extract. In conclusion, the ratio *R* did indeed increase in the leather extracts, due to the leather fraction delivering additional cattle mtDNA to that issued from the glue fraction. The final results from the Q-PCR experiments proved that the leather used by Erard to cover the hammer heads in his piano were indeed from cattle hide. This novel experimental strategy and analysis methodology based on mtDNA quantity analysis can also be applied to various types of complex processed samples, paving the way to a systematic molecular analysis of historical musical instruments or any other historical remains.

## 4. Ancient mtDNA and Past Human Diets

The functional role of mitochondria in metabolism is another key aspect for analyzing ancient mtDNA to trace the origin of a population [28]. Several studies have concentrated on the hypervariable segment I and hypervariable segment II polymorphic regions in the displacement loop (D-loops) of mtDNA to trace the origins of populations [29,30]. Through evolution, the human genome, including mitochondrial genome, has acquired genetic adaptations to its diet, which can be used to map evolutionary processes in space and time. Studies on ancient DNA have provided a strong link between food availability and nutritional content and diversity in natural populations [31,32,33,34,35]. This has brought substantial focus on mitochondrial DNA as a marker of dietary adaptation of ancient generations. There are several advantages of using mtDNA in these studies. The ratio of mtDNA to nuclear copies is high, which aids in its isolation and extraction from ancient samples. mtDNA is haploid in nature, and thus should evolve four times faster than the average nuclear gene. Hence, mtDNA can be used to track divergence in very closely related taxa and even within species [36]. Another advantage for mtDNA is its minimal recombination; although it was recently shown that recombination in human mitochondrial DNA is possible, the frequency of these processes is very limited compared to nuclear DNA [37,38]. The functional role for mitochondria in metabolism has been well studied, and thus its role in diet and nutrition is well established. The mitochondria are the site of essential metabolic processes in the cell needed to generate energy from proteins, carbohydrates, and fatty acids [39,40]. The main function of mitochondria is to couple respiration and substrate oxidation to generate ATP [41,42]. The human mitochondrial genome encodes 13 proteins of the electron transport chain [6]. Studies have shown that diet can affect mitochondrial gene expression [43]. This led to a substantial increase in studies focusing on mtDNA polymorphisms in populations and across distant geographical areas as a pivotal tool in evolutionary genetics. In 2015, a study by Malmström et al. [44] using deep-sequencing of ancient mtDNA D-loops from Neolithic samples discovered genetic diversity between farmer and hunter-gatherer groups in Neolithic and Mesolithic Europe. In this study, mtDNA hypervariable region 1 (HVS1) analysis was done on 49 Neolithic individuals recovered in different archaeological contexts in present-day Sweden. Ten of the samples were recovered from bones and teeth samples from Megalithic passage graves associated with the TRB culture (funnel beaker culture, TRB from German Trichterbecherkultur), and dated to approximately 4500 years before present YBP. TRB culture is believed to have been the first farming society in northern Europe and Scandinavia. Thirty seven hunter-gatherer samples were obtained from the Pitted Ware Cultural complex (PWC) contexts on islands on the Baltic Sea and dated approximately to 5200–4400 YBP. Two samples representing Battle Axe Culture (BAC) burial, a Scandinavian variant of the Corded Ware complex, were from a grave containing a male, a female, and an infant, found in Linköping, Östergötland and dated to 4800–4100 YBP. After sequencing, the consensus sequences from these samples were merged with previously published Mesolithic and Neolithic sequences of six groups from three different geographical areas: Scandinavian Neolithic farmers and hunter-gatherers [44], Central European Neolithic farmers [45] and Mesolithic hunter-gatherers from 13,400 before Christ (BC) to 2300 BC [46], Iberian Neolithic farmers from ca. 6000 YBP [47,48], and Mesolithic hunter–gatherers from ca. 6000–7000 YBP [47,49]. The data from this study showed that mtDNA of the farmers of northern Europe and Scandinavia is closely linked to that of early Central European, but not to the concurrent PWC hunter-gatherers. More importantly, the study showed the existence of diversity in mtDNA between groups with different subsistence patterns (hunter-gatherers and farmers), suggesting a potential role for genetic adaptations to food and dietary-related behaviors. However, since both groups have different ancestries, it can be strongly argued that the mtDNA variation is solely due to genetic variations between their antecedents, without the influence of their dietary adaptations. In another study by Morris et al. [50] in 2014, analysis of the first complete ancient Southern Africa Khoesan mtDNA showed that ancient maternal human lineages were present in Southern African people living near coastal areas prior to the arrival of southward migrating farmers. The ancient mtDNA sequences have provided evidence in support of osteological studies on the discovered Khoesan remains to show the absence of tooth decay and excessive occlusal wear and suggesting a diet typical of hunter-gatherer lifestyle [50]. Another recent study by Hamilton-Brehm et al. [51] screened human mitochondrial DNA sequences from the quids found in Mule Spring Rockshelter, Nevada, USA, dating back to late Holocene Epoch around 1000 YBP. Squids are the expectorated indigestible fibers from the chewed agave or yucca root consumed by local inhabitants. The haplogroups from this study belong to the major pan-American lineages, mainly haplogroup C1c [52,53]. C1c and C1c2 subgroups resemble native American crossing Beringia [52,54]. The results suggested that dominance of haplogroup C1 in Mule Spring Rockshelter, Nevada, is due to maternal lineages of the C1c and C1c2 subgroups that routinely visited that area, or that individuals in this type deposited a higher number of squids than other visitors. The interest in deciphering our ancestors’ dietary lifestyle has motivated researchers to study ancient parasites colonizing the human intestine [55]. Ancient mitochondrial DNA from human-specific parasites is used to track ancient migrations and study the population genetics, diets, habits, and health of ancient civilizations (for a detailed review on this topic, see Reference [56]). In a recent study by Søe et al. [57], shotgun sequencing was performed on ancient parasites found in samples from Northern Europe and the Middle East (500 BC–1700 AD) [57]. Filtered reads were screened against a published mitochondrial reference database of *Ascaris lumbricoides* and *Trichuris trichiura* and assigned to the most recent common ancestor. The study identified not only ancient parasites that are transmitted between humans, but also helminths that have domestic animals or fish as definitive hosts, suggesting dietary adaptations to the geographical distribution of these populations. Taken together, these results suggest that ancient mtDNA is a powerful option for investigation of the diet, lifestyle, and population history of ancient generations. The superiority of mtDNA over nuclear DNA in tracing hominin evolution will undoubtedly pave the way for more research in the future. In this sense, it will be appropriate to say that it will be the end of the beginning for the use of mtDNA as a marker for revealing past diet.

## 5. Ancient Mitochondrial DNA: A Powerful Tool to Reveal Mysteries in Mummies

“Ancient nuclear DNA” has routinely been used to analyze subfossils and their evolutionary histories. However, the use of ancient nuclear DNA for studying Egyptian mummies has been highly debatable. There is one group of scientists that believe in the use of PCR-based techniques for ancient nuclear DNA, while there is another group of scientists that do not believe in such findings [58,59,60,61,62]. This group of scientists argues that nuclear DNA is prone to degradation, especially in the hot and humid climate of Egypt. Therefore, such old mummies may not have any good quality nuclear material available for analysis, and the results obtained from this DNA could be due to contamination [58,59,60,61,62]. There are many published articles that accept and refute such hypotheses. While these controversies are ongoing, we would like to focus this part of the review on the alternative system that has been used for genetic analysis of mummies: that is, mitochondrial DNA. There is a plethora of information available related to anthropology and evolution where mtDNA has been found to be very instrumental [58]. The relevance of the mitogenome for such studies might be due to the occurrence of mtDNA in high copy numbers and its intrinsic property of less degradation as compared to nuclear DNA [58]. It is interesting to point out that the rate of mutation in the mitogenome (around 2–4% per million years) is much higher than in the nuclear genome, due to the absence of proofreading enzymes for mtDNA [63,64]. To obtain more reliable data, new techniques such as next-generation sequencing of the mitochondrial genome have been introduced [65,66]. For the first time, deep sequencing was used on mummified samples that were obtained from 806 BC–124 AD, where the ancestral lineage of ancient Egyptians was traced using mtDNA [65]. In addition to the DNA of humans, the DNA of *Plasmodium* and *Toxoplasmosis* was obtained in the sequencing library, which shows the presence of corresponding pathogens in mummies [65]. However, there are more failures than successes in this field of research. One of the drawbacks in next-generation sequencing is a very low abundance of mitochondrial DNA (less than 0.06%) in sequencing libraries [67]. Despite these problems, mitochondrial DNA has been successfully studied for crocodile and cat mummies [68,69]. In sacred ibis mummies, the enrichment of mtDNA has been achieved by a targeted hybridization method using biotinylated RNA baits, which led to 4705-fold enrichment [67]. In the last decade or so, the full mitogenomes of the mummies were sequenced and their specific haplogroups were identified [70]. The oldest complete human mitogenome that was sequenced belonged to a prehistoric European [71]. In this study, the Tyrolean Iceman (lived around 5350–5100 years ago) corpse’s mtDNA was amplified and sequenced. When compared with related extant lineages, it was found that Iceman belonged to the mitochondrial haplogroup K1 [71]. In Native American mummies, the first hypervariable region (HSV-1) of mtDNA was targeted, including findings on Kwäday Dän Ts’ìch, obtained from a glacier in British Columbia, and an Inca child mummy. The outcome of this study showed that the mtDNA HSV-1 and other mtDNA SNPs point towards the allocation of these mummies to haplogroups C and D [72,73]. Furthermore, Antonio Salas and colleagues analyzed the full mitogenome of a 7 year old child, who was a victim of an Inca child ritual around 500 years ago, and this mummy was found in the Argentinian province of Mendoza. The mtDNA analysis showed that the mummy belongs to the C1bi haplogroup, which corresponds to the present-day haplogroup of Peru and Bolivia [70]. Schuenemann et al. obtained mtDNA from 90 mummies, dated within a span of 1300 years from 1380 BC to 425 AD. Surprisingly, ancient Egyptians’ mtDNA matches more with ancient Europeans and Anatolians than modern Egyptians [66]. More importantly, the nuclear DNA showed poor stability as compared to mtDNA, which was evident by the high ratio of mtDNA and nuclear DNA (around 18,000) [66]. Notably, out of more than 160 samples, only three of gave a satisfactory SNPs result. A 111 year old mystery of royal mummies was recently solved using next-generation sequencing of mitochondrial DNA. In 1907, two mummies around 4000 years old were found in the south of Cairo, Egypt [74]. The anatomy and skin pigmentation of these mummies concluded that they were not related to each other. However, the follow up on their mtDNA and Y chromosome DNA later found that the two mummies had the same mother but different fathers. Therefore, these two mummies were not brothers but half-brothers [74]. Overall, it seems that the next generation sequencing of mtDNA has boosted the field of mummies, and has paved the path for exploring it further. However, this is just the beginning, and more extensive studies are needed to verify the findings obtained through archeology, literature, anatomy, etc. DNA sequencing of mtDNA is indeed a very powerful source of information. It should be applied with other new technologies to give impetus to the research of subfossils and mummies, which has been mostly elusive until now.

## 6. mtDNA as a Key Element to Uncover Geographical Domestication Origins and Lineages Relationships

Animal domestication is considered an important step of human civilization that marked the early signs of development of past people, especially their technological and social development. Studying animal domestication, mtDNA has always been the key element to understanding the geographical origins of domestication and the lineages relationships as well. In the following paragraphs, specific animals will be discussed in detail to include sheep, pigs, chicken, goat, cattle, dogs, and cats, outlining studies and analyses that better distinguish and detect the lineages between these animals.

### 6.1. Sheep

Domesticated sheep have represented a significant part of human civilization, especially regarding food, wool, and hide productions, since the Neolithic Agricultural revolution (8000–9000 years ago). It has been suggested that numerous wild sheep species, with confused taxonomy, are the ancestors of modern domestic sheep *Ovis aries* [75]. Recent studies based on mtDNA variation identified five domestic sheep haplogroups (HA, HB, HC, HD, and HE) [76]. Haplogroups HD and HE are the rarest (Caucasus and Turkey) [76]. Haplogroup HC has also a restricted distribution located in the Fertile Cresent, Caucasus, and the Iberian Peninsula [77]. 

HA and HB were the most frequently identified haplogroups in an interesting study authored by Hiendleder et al. [78]. In this study, the authors analyzed mitochondrial DNA from 243 sheep of different breeds from Kazakhstan, Germany, and Turkmenia. In the same study, mtDNA was obtained from preserved sheep tissue samples that were collected from Moscow State University and Justus-Liebig University, Giessen. The mtDNA analysis showed twenty haplotypes in three major phylogenetic groups: urial/argali, mouflon/domestic, and domestic sheep. The results of Hiendleder et al. indicate two major domestic sheep lineages from the branches that contain mouflon and domestic sheep. The first lineage, called European lineage, contains the majority of haplotypes detected among European domestic sheep. The second lineage, called Asian lineage, consists of haplotypes found in central Asian and some European domestic sheep. In addition, Hiendleder et al. results suggest that modern domestic sheep—which are mostly HA and HB haplogroups—derived from an additional unidentified wild ancestor, other than the urial and argali groups. 

### 6.2. Pig

The findings of Caliebe et al. [79] show that around approximately 4500 BC, domesticated pigs bearing near Eastern haplotypes appeared in northern Europe, based on the ancient mitochondrial DNA haplotype data from 116 Neolithic Sus specimens. A computational model was developed and used by the authors in order to identify the origin of domestic pigs through mitochondrial haplotypes. Their computational assessment suggests that between 5000 and 4000 BC, domesticated animals from the south of central Europe were the origin of almost all the matrilineal lines in the north. In contrast, this study showed that during the period 4000–3000 BC, the majority of the northern European lineage domestic pigs originated from local wild boars, with little contribution by imports from the south.

### 6.3. Chicken

Archaeological evidence suggest that domestication of the chicken *Gallus gallus* occurred in different geographical sites in India and China at least 5400 years ago [80]. In a recent study, Lorenzo et al. assessed mitochondrial DNA to determine the origin of domestic chicken [81]. Their research confirmed that, thanks to mtDNA analysis, most of the studies on chicken domestication show that Indochinese Red Junglefowl subspecies *Gallus gallus gallus* is the primary maternal ancestor of the domestic chicken *Gallus gallus domesticus*. Lorenzo et al. also concluded that genetic contributions from other junglefowl, like the Grey Junglefowl *Gallus sonnerati* and the Ceylon Junglefowl *Gallus lafayeti*, should also be considered.

### 6.4. Goat

Previous mtDNA-based studies have indicated that the diversity of domesticated goats *Capra hircus* in Europe is a subset of the wild goat *Capra aegagrus* [82], which is found in the Zagros and Taurus mountains, where the earliest archaeological evidence (11,000–10,000 YBP) for goat domestication was revealed [83]. Other mtDNA-based studies revealed that domestic goats have highly divergent maternal lineages (A, B, C, D, and E) [84]. In a detailed study based on published mtDNA data of *C. hircus* and *C. aegagrus*, Gerbault et al. [83] used coalescent simulations and approximate Bayesian computation to determine if domestic goats descend from populations that were distinct prior to domestication. Their results show a potential bidirectional migration between the wild and domesticated population. In other words, their results indicate that wild and domestic goats are most likely to descend from a single ancestral wild population 11,500 YBP.

### 6.5. Cattle

From the silver mining in medieval times until the end of World War II, the Red Mountain Cattle (known in German as Rotes Höhenvieh, RHV) breed, which is an important native ancient breed from the middle of Europe, was raised for milk, meat, and as a draught animal [85]. When this breed was close to extinction around the 1980s, only sperm from a single purebred bull and a few cows were available for breeding. Ludwig et al. [85] sequenced complete mitochondrial genomes from six RHV cows, representing founder lineages. Ludwig and his colleagues found a unique RHV mitochondrial gene pool, including six mitochondrial genotypes. None of those genotypes has been found in any other cattle breed to date. Their findings offer great possibilities for the conservation of this endangered breed, characterized by an excellent calving record with sufficient milk production.

### 6.6. Dog

The domestic dog (*Canis familiaris*) is thought to be the earliest domesticated animal [86]. Archaeological evidence from South West Asia indicates that domestic dogs existed by 11,500 YBP [87]. A study by Ardalan et al. [88] performed a detailed analysis of mtDNA diversity, based on extensive samples of indigenous dogs from the Persian Plateau, Anatolia, and the so-called Fertile Crescent region extending from Persian Gulf to Eastern Mediterranean coast. The Ardalan et al. study showed that only 5 of the total 10 principal sub-haplogroups belonging to a universal mtDNA gene pool were found in South West Asia. Instead, specimens from the Asian region south of the Yangtze River (ASY) identified all 10 principal sub-haplogroups, suggesting this to be the center of origin for modern dogs.

Frantz et al. [89] explained the origins of dogs, which are difficult to understand geographically. They sequenced mtDNA from 59 ancient European dog samples (14,000 to 3000 years ago) and a complete nuclear genome of a late Neolithic dog from Ireland (4800 YBP). To evaluate the consistency between their results and the archaeological record, Frantz et al. collected evidence of the earliest dog remains across Eurasia. This study concluded that dog remains should be found in Central Eurasia (8000 years and older), as, so far, dog remains have been recovered from old sites in Eastern and Western Eurasia only (15,000 YBP to 12,500 YBP). Based on ancient and modern mitochondrial DNA sequence analysis, Frantz et al. showed a significant gap in mitochondrial haplotype frequencies in Europe. The majority of ancient European dogs belonged to either haplogroup C or D, while modern European dogs retain sequences within haplogroups A or B. In addition, results from this study suggest that dogs may have been domesticated independently in Eastern and Western Eurasia from distinct wolf populations, and that dogs from East Eurasia reached Europe with human migration and were partially replaced with European Paleolithic dogs.

### 6.7. Cat

Information about the domestication of cats and their dispersal around the world is limited. As per Ottoni et al. [90], ancient mtDNA analysis of cat remains from geographic and archeological sites shows that both Near Eastern and Egyptian populations of *Felis silvestris lybica* have shared the gene pool of the domestic cat at various periods. Based on the reestablished mtDNA phylogeny of Ottini et al., there are five geographically partitioned subspecies, identified as *Felis silvestris silvestris*, *Felis silvestris lybica*, *Felis silvestris ornata*, *Felis silvestris cafra*, and *Felis silvestris bieti*. In the study of Driscoll et al. [91], analysis of nuclear short tandem repeats (STR) and mitochondrial DNA genomes of wild and domestic cats revealed that only North African/Southwest Asian *F. s. lybica*, was domesticated [91].

## 7. Ancient Trade Routes and Mitogenome Analysis

In this section, we are going to focus on the exceptional power of mitochondrial DNA analysis, which can trace maternal ancestry back hundreds of thousands of years, and is having a great impact on the studies of both modern and ancient remains to determine the absolute truth behind documented ancient trade routes.

### 7.1. Ancient Indo-Roman Maritime Trade Route

Historically, it was documented that Indo-Roman trade through the Malabar port was intensified where precious items like gold, pearls, and textiles were imported from Tamil Nadu of South India [92]. In a recent study by Palanichamy et al., trade links between Mesopotamian civilization and the Indian subcontinent were evaluated using a large mtDNA data set [93]. This study was inspired by previous research conducted by Witas et al., who were able to analyze mtDNA obtained from skeletal remains found in Mesopotamian civilization sites and dated between 2500 BC and 500 AD. The results of Witas et al. suggested that the skeletal remains from Mesopotamia belonged to people with a genetic affinity with the populations living in the Indian subcontinent [94]. To identify the genetic link between ancient Mesopotamians and Indian populations, Palanichamy et al. compared the ancient Mesopotamian mitochondrial haplotype with 15,751 modern mitochondrial DNAs, representing all major populations of India (11,432 from the literature and 4319 from their study). The results clearly indicate that the sequence of the ancient Mesopotamian MK 11G 107 haplotype was matched within the populations living in a restricted area of Dindigul district of Tamil Nadu. This genetic affinity explains the Tamil–Mesopotamian connection, which supports the historical documentation of the trade between Rome and south India during the first centuries AD.

### 7.2. Ancient Phoenician Trade Routes and Genetic Affinity of Ancient Phoenician with a European Haplogroup

Arwad, Sidon, Tyre, and Byblos, main Phoenician coastal cities, located in what is now Lebanon and southern Syria, have been continuously occupied, and therefore seldom subjected to major archaeological excavations. The voyaging Phoenicians were famous for their trading skills, and it is clearly documented that they reached the Atlantic costs of Spain and Morocco [95]. The Phoenicians established trade posts across the different Mediterranean cities, and Carthage in North Africa was a Phoenician settlement which was destroyed by the Romans [96]. Skeletal remains of a young Phoenician man dated late 6th century BC were discovered in Carthage. In a study conducted by Matisoo-Smith et al. [97], the only element of molecular analysis was the mitochondrial DNA. In their study, the ancient Phoenician mtDNA was compared with 47 modern Lebanese mtDNAs, ancient Levantine mtDNA, and European mtDNA. The complete mitochondrial genome retrieved from the young Phoenician man determined that he indeed carried a European haplogroup. Matisoo-Smith et al. definitively linked his maternal ancestry to Phoenician-influenced locations somewhere along the Northern Mediterranean coast, the islands of the Mediterranean, or the Iberian Peninsula. This amazing result provided the first direct ancient DNA proof of a Phoenician individual, along with being the earliest evidence of a European mitochondrial haplogroup, U5b2c1, in North Africa.

## 8. Conclusions

In this review, we have discussed the role of mitochondrial DNA in studying diverse biological processes. The most important advantages of using mtDNA are its intrinsic ability to resist degradation and its high copy number inside the cell as compared to nuclear DNA (nuDNA). Each cell contains around 1000 mitochondria, and there are 2–10 copies of the mtDNA per mitochondrion [98]. Thus, the amount of mtDNA available from a sample is usually high. However, the use of mtDNA also has some limitations and shortcomings. In this section, we have outlined the most relevant limitations. 

### 8.1. MtDNA and Its Limitation in Deciphering Criminal Cases

NuDNA is bi-parentally inherited and is usually unique for an individual, except in the case of identical twins. The key problem in using mtDNA in criminal cases is that it is maternally inherited and usually does not undergo any recombination [99,100]. In the absence of a recombination event, the offspring is highly likely to inherit the same DNA from the mother. Since the mother, siblings, or any other maternally associated individuals will have the same mtDNA profile, it is not possible for investigators to uniquely identify the subject [101]. There is also a problem of contamination of ancient mtDNA with modern DNA, which can lead to artefacts and thus to misleading results [102]. Several of the pioneering studies in 1990s and early 2000 were seen as breakthroughs in the analysis of subfossil remains. It is now known that studies based on mitochondrial hypervariable region 1 (HVR1) are mostly artefacts due to its contamination with modern DNA [103]. Any study on fossil specimens based on ancient DNA should thus be received with caution, and must be verified with other independent techniques. Moreover, the study of mtDNA is also expensive and time consuming as compared to nuDNA [104]. Despite these downfalls, it is important to point out that mtDNA profiling is an outstanding technique for such cases where there is an insufficient amount of nuDNA available, or its quality is unsuitable to make any inference [36].

### 8.2. Molecular Damage in Postmortem mtDNA

Population genetics study often requires the use of ancient DNA. These studies are highly informative, but they should be interpreted with caution, because postmortem DNA damage is a factor that is often not taken into consideration. Due to postmortem DNA degradation in ancient samples, there can be extreme discrepancies in the analysis [105]. In a study by Binladen et al., 23 ancient samples were investigated for their mtDNA and nuDNA. Although there were significant differences in the copy numbers of the mtDNA versus nuDNA, there was no difference in the miscoding lesion damage recorded between the two types of DNA [106]. In another study, it was shown that, in human mtDNA, the control regions of human HVR1 that are considered a hot spot of postmortem damage correspond to regions of high mutation rates [107]. Therefore, to identify the incorrect assignment of haplogroups, a simplified method has been introduced based on postmortem damage [107]. 

### 8.3. Diversity of mtDNA

MtDNA has been shown to be an ideal marker for molecular diversity. The reasons for this are its ability to be clonally inherited, neutral or near neutral molecular evolution, and that its constant accumulation of neutral or slightly deleterious mutations with time enables accurate dating of samples [108]. The diversity of mtDNA existing at the individual level is known as population diversity, and that which exists at the cellular level is known as heteroplasmy. Due to minimal recombination in mtDNA, any polymorphism that arises can be precisely located in the phylogenetic tree. This is evident in the form of haplotypes and the corresponding haplogroup. Although mtDNA is usually involved in neutral or near neutral molecular evolution, recent evidence points towards its role in diverse physiological and pathological conditions such as ageing, fertility, disease susceptibility, etc. [109,110,111,112]. 

Autosomal DNA contains the most abundant polymorphisms in human populations. Single nucleotide variants have an estimated mutation rate of 10^−7^ to 10^−8^ per generation [113]. Unlike this slow mutation rate, the mutation rate of microsatellite systems (repeated DNA sequence) is very high, in the range of 10^−3^ per generation [114]. On the contrary, the mtDNA mutation rate is in the range of 2–3 × 10^−7^ per generation [29]. Antithetical to the mode of inheritance of mtDNA, which is maternally inherited, Y chromosome DNA is paternally inherited. Thus, Y DNA polymorphisms are very rare [115,116]. Due to this reason, Y chromosome has been an excellent genetic marker to study human evolution, expansion, and migration. Recent data also suggest that both positive and negative selection act on the Y chromosome and thus influence Y haplotype distribution in human populations [117]. In future studies, it will be interesting to compare the molecular diversity of mtDNA, autosomal markers, and Y chromosome genetic markers for a particular dataset. This will be helpful to understand the respective benefits of one over the other. 
